# Absolute monocyte count has a diagnostic role in distinguishing tumor marker-negative TGCT from benign testicular tumor via CCL2 regulation

**DOI:** 10.1097/MD.0000000000034114

**Published:** 2023-06-23

**Authors:** Li Cao, Qinzheng Chang, Jiajia Sun, Shuo Pang, Yidong Fan, Jikai Liu

**Affiliations:** a Department of Urology, Qilu Hospital of Shandong University, Jinan, Shandong, P.R. China.

**Keywords:** AMC, CCL2, MLR, TGCT, tumor marker

## Abstract

Clinically, for testicular tumor patients with negative tumor markers, how to distinguish the malignant from the benign is a difficult problem. This study aimed to assess the clinical significance of the absolute monocyte count (AMC) in differential diagnosis of testicular germ cell tumor with stage S0 (TGCT^S0^) and benign testicular tumor. In this retrospective single-center study, a total of 90 patients newly diagnosed with benign testicular tumor or TGCT^S0^ were reviewed. All patients received surgical intervention as the primary treatment method. AMC and other clinicopathological parameters were analyzed. Receiver operating characteristic (ROC) curves were constructed to assess the diagnostic power of investigated parameters, and to determine the optimal cutoff values. Kaplan–Meier curve analysis was used to study the survival of patients with TGCT^S0^. qRT-PCR and immunohistochemistry (IHC) were performed to examine the expression of C-C motif chemokine ligand 2 (CCL2) mRNA and protein respectively. Differential gene expression and functional enrichment analysis were performed using Gene Expression Omnibus and the Cancer Genome Atlas databases. The mean preoperative AMC in patients with TGCT^S0^ was significantly higher than that in patients with benign testicular tumor (*P* = .020). AMC > 0.485*10^9/L was identified to be associated with the presence of TGCT^S0^ (hazard ratio [HR] = 3.074, *P* = .026), and patients with higher AMC level had worse progression free survival (PFS) (*P* = .047). Furthermore, AMC combined with lactate dehydrogenase (LDH) achieved a better diagnostic efficacy for TGCT^S0^ (area under curve [AUC] = 0.695). Tumor-associated macrophages (TAMs) signature gene CCL2 was highly expressed in TGCT compared with normal testicular tissue. Functional enrichment analysis showed that CCL2 is closely involved in the Extracellular Matrix Organization pathway and positively correlated with the expression of various matrix metalloproteinases (MMPs). Elevated AMC may serve as a predictor of higher risk of TGCT^S0^, and CCL2 mediated TAMs infiltration and MMPs secretion is essential for the tumorigenesis of TGCT.

## 1. Introduction

Testicular cancer (TC) is the most frequent solid malignancy occurring in males between the ages of 15 and 40 years, accounting for 1% to 2% of all malignant tumors in men, with a steadily rising incidence for the past few decades.^[1,2]^ Testicular germ cell tumor (TGCT) account for 95% of TC, while other malignancies include lymphoma, metastatic tumors, and residual adrenal tumors. Histopathologically, 55~60% of all TGCTs are classified as seminomas, and the remaining cases as non-seminomas, including teratomas, yolk sac tumors, embryonal carcinomas, choriocarcinomas, and mixed GCTs.^[3,4]^ The benign testicular lesions are mainly sex cord-stromal tumors, epidermoid cysts, dermoid cysts, adenomatoid tumors, angioma, testicular tuberculosis, orchitis, etc.^[5]^ The traditional treatment for TC is radical orchiectomy, but for benign testicular tumors, radical orchiectomy may result in the loss of excessive testicular tissue in these patients, which is physically and psychologically traumatic for men of any age, especially men of reproductive age. It has been documented that the removal of 1 testicle reduces spermatogenesis in men.^[6]^

In clinical practice, ultrasound, MRI and serum tumor marker detection are usually the preferred methods to identify testicular lesions. At present, serum markers commonly used include α-Fetoprotein, beta subunit of human chorionic gonadotropin (β-HCG) and lactate dehydrogenase (LDH), which play an important role in the diagnosis, staging and prognosis of testicular tumor. However, β-HCG and LDH are elevated in only 28% and 29.1% of seminomas, while α-Fetoprotein, β-HCG, and LDH are elevated in 60.1%, 53%, and 38.7% of non-seminomas, respectively.^[7,8]^ LDH is a tumor marker with low specificity since it may be elevated due to a number of reasons, and elevation is mostly seen in patients with advanced testicular tumor. Overall, serum tumor markers have a low sensitivity and specificity, especially in seminoma, such that despite tumor markers being negative, there are still a considerable number of patients with the possibility of TGCT, which classified as stage S0 according to TNM staging issued by UICC.^[9]^

As for the rapid intraoperative pathological examination, most scholars believe that it is the “gold standard” to judge the benign and malignant tumor.^[10]^ However, some scholars believe that rapid intraoperative pathological examination will destroy the blood-testosterone barrier, leading to the entry of hidden sperm antigens into the blood, triggering the production of anti-sperm antibodies (ASA), thus affecting the reproductive function of the remaining testis.^[11]^ In the context of personalized medicine, new serum markers will provide clinicians with a better way to characterize tumor diseases. Studies have found that inflammatory response plays a very important role in the development of tumors, and is an important influencing factor in the tumor microenvironment.^[12–17]^ Peripheral absolute monocyte count (AMC) has become an important indicator of tumor diagnosis and prognosis.^[18–21]^ However, clinical utility of AMC, regarding the diagnosis of testicular lesions, has not been studied yet. Therefore, we aim to assess the performance of AMC in differential diagnosis of testicular benign tumors and TGCT with stage S0 (TGCT^S0^) in this study.

## 2. Materials and methods

### 2.1. Patients

A review of medical records was performed for 168 patients who were newly diagnosed with testicular tumors and received surgical management at the Department of Urology, Qilu Hospital of Shandong University, between January 2006 and December 2022. Prior to participation, the following criteria were used to exclude patients: Coexisting any other malignancy; Recurrent testicular neoplasms; Testicular inflammatory diseases, including acute orchitis, testicular abscess, testicular tuberculosis; Preoperative radiotherapy or chemotherapy was performed; Inadequacy of surgically removed tissue samples for complete histopathological evaluation. The study was conducted according to the guidelines of the Declaration of Helsinki and approved by the Ethics Committee of Qilu Hospital of Shandong University.

**Figure 1.**
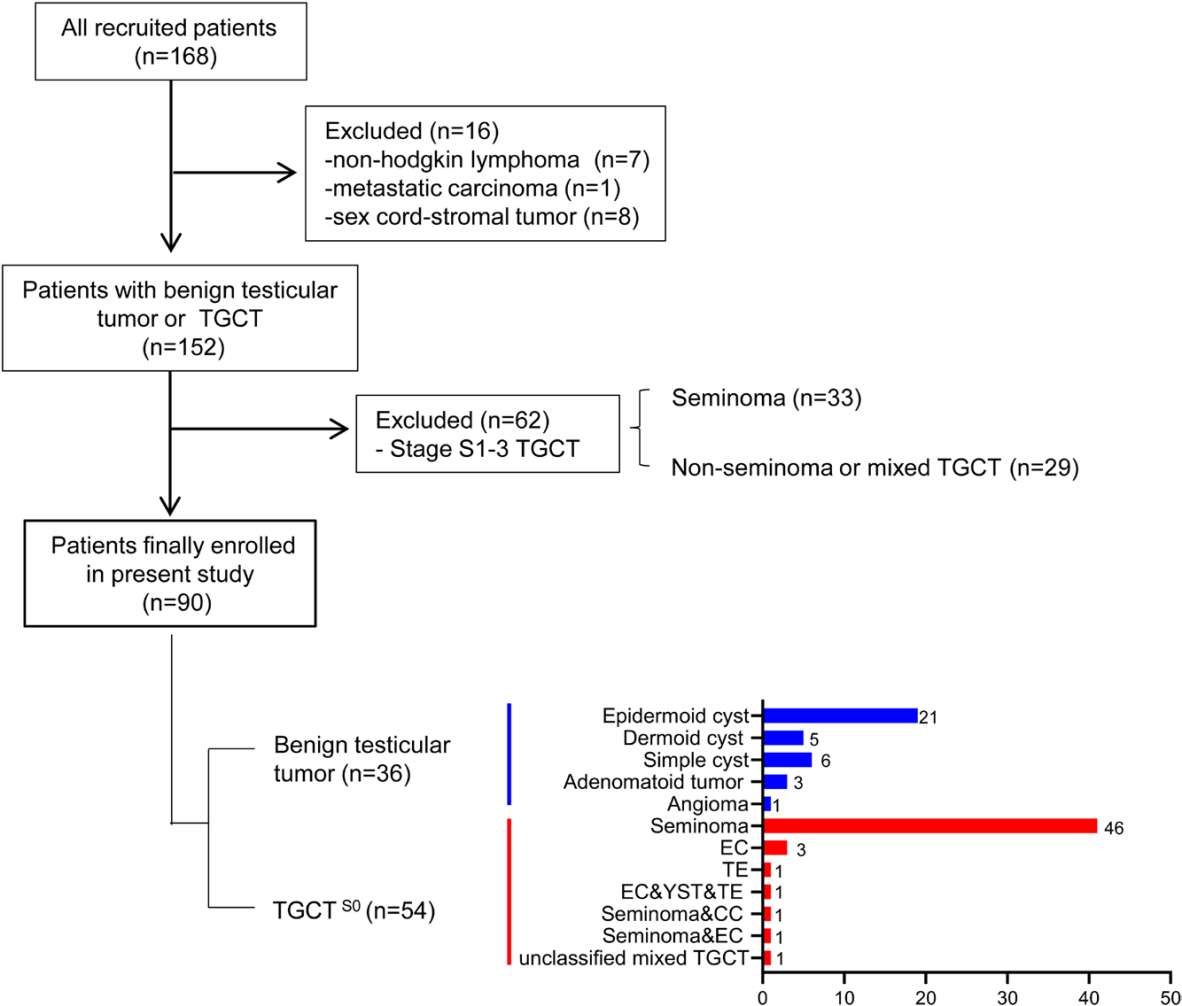
Patients included in this study. CC = choriocarcinoma, EC = Embryonal carcinoma, TE = teratoma, TGCT^S0^ = testicular germ cell tumor with stage S0, YST = yolk sac tumor.

As shown in Figure 1, of the 168 patients involved in the study, we first excluded 8 other types of malignancy (7 non-Hodgkin lymphoma and 1 metastatic cancer) and 8 sex cord-stromal tumor. Among the remaining TGCT patients, 62 patients with stage S1 to S3 were eliminated, and 90 patients were finally enrolled in this statistical study, including 36 patients with benign tumors and 54 patients with TGCT^S0^. Histologically, among the benign tumors, 21 cases were epidermoid cysts, 5 cases were dermoid cysts, 6 cases were simple cysts, 3 cases were adenomatoid tumors and 1 case was angioma. For the patients with TGCT^S0^, 46 cases exhibited seminoma and 8 cases showed non-seminoma, including 3 cases with embryonal carcinoma including 2 cases with embryonal carcinoma, 1 case with teratoma and 5 cases with mixed TGCT.

### 2.2. Data collection

Clinical data including patient age at the time of diagnosis, smoking history, routine blood examination results, and tumor characteristics were obtained from the electronic patient records at our institution.

### 2.3. Bioinformatic analysis of clinical data

Gene Expression Omnibus datasets (GSE3218 and GSE8607) were used for gene expression analysis. mRNA expression and survival data of TGCT cases were obtained from The Cancer Genome Atlas Data Portal. Cell proportion analysis of macrophages in tissues was obtained from Gene Expression Profiling Interactive Analysis 2021 (GEPIA2021).^[22]^

### 2.4. quantitative RT-PCR (qRT-PCR)

qRT-PCR analysis was conducted on a Quant Studio 5 (Applied Biosystems) with SYBR Green Premix Pro Taq HS qPCR Kit Accurate Biotechnology ((Hunan) CO., LTD, ChangSha, China). The reaction conditions were 95°C for 30 seconds, 40 cycles of 95°C for 5 seconds, 60°C for 30 seconds, and 72°C for 15 seconds, 60°C for 1 minute, 95°C for 1 second. TRIzol reagent (Thermo Fisher Scientific, Waltham, MA) was used to extract total RNA from benign testicular tumor and TGCT^S0^ tissues. The primers were C-C motif chemokine ligand 2 (CCL2), 5′-CAGCCAGATGCAATCAATGCC-3’(forward) and 5’-TGGAATCCTGAACCCACTTCT-3’(reverse); GAPDH, 5’-CCAGCCGAGCCACATCGCTC-3’(forward) and 5’-ATGAGCCCCAGCCTTCTCCAT-3′ (reverse).

### 2.5. Immunohistochemistry (IHC)

Immunohistochemical studies were performed on formalin-fixed and paraffin-embedded tissue sections. The benign testicular tumor and TGCT^S0^ tissues were collected in formaldehyde, embedded in paraffin and cut into 5 µm thick sections. The antigen retrieval was performed with citrate buffer pH 9.0 (ZLI9069; ZSGB‑BIO, Beijing, China). Slides were incubated at 37°C for 1 hour with anti-CCL2 (A7277; ABclonal, Wuhan, China, 1:100) 4°C overnight. The histological sections were then stained with the DAB Kit (PV‑9000; ZSGB‑BIO, Beijing, China) to examine the expression of CCL2 in each slide.

### 2.6. Statistical analysis

Values are presented as mean ± standard deviation (SD). The Mann–Whitney *U* test were used to compare the parameters between 2 groups. To determine the optimal threshold values for differential diagnosis, receiver operating characteristic (ROC) curve analysis was performed. The value of parameters in discriminating TGCT^S0^ was further assessed using logistic regression analyses. Relative risks were estimated using hazard ratio (HR) of the logistic analysis with 95% confidence interval (CI). The Spearman correlation test was used to test relation between numerical variables. Survival curves were generated with the Kaplan–Meier method. A 2 sided *P* value <.05 was regarded as statistically significant. All analyses were performed using the Statistical Package for Social Sciences version 23.0 (SPSS Inc., Chicago, IL).

## 3. Results

### 3.1. Clinical features of study population

The present study included 90 patients with newly diagnosed testicular tumor. Among these, 36 were benign tumors and 54 were TGCT^S0^, with a mean age at diagnosis of 32.00 ± 14.89 years (median: 27.5 years, range: 16–67 years) and 38.67 ± 12.91 years (median: 36 years, range: 21–77 years), respectively. Two groups had similar rates of smoking history (22.22% vs 24.07%), and tumors are both more likely to occur on the left side (55.56% vs 51.85%). Of 36 benign tumors, 15 case (41.67%) underwent orchidectomy and the rest received partial orchidectomy, while all malignant tumors were treated with radical testicular resection. The average size of benign tumors and TGCT^S0^ was 2.82 ± 1.91 cm (median 2.45 cm, range: 0.9–11.0 cm) and 4.36 ± 2.13 cm (median 4.0 cm, range: 0.5–10.0 cm), respectively. In addition, the absolute counts of peripheral blood cells, including neutrophil, lymphocyte, monocyte and platelet, were obtained from all patients, as shown in Table 1. Patients with TGCT^S0^ showed higher AMC than patients with benign tumor (*P* = .020). What’s more, among the serum indicators, LDH level was significant higher in TGCT^S0^ compared to benign tumor (*P* = .035, Table 1).

**Table 1:** Comparison of clinical characteristics between benign testicular tumor and TGCT^S0^ in study patients.

Characteristics	Benign	TGCT^S0^	*P*
	N = 36 (40%)	N = 54 (60%)	
Age (yr)			.002
Mean ± SD	32.00 ± 14.89	38.67 ± 12.91	
Median (range)	27.5 (16–67)	36 (21–77)	
Smoking history			
Never	28 (77.78%)	41 (75.93%)	
Ever	8 (22.22%)	13 (24.07%)	
Side			
Left	20 (55.56%)	28 (51.85%)	
Right	16 (44.44%)	26 (48.15%)	
Surgery procedure			
Orchidectomy	15 (41.67%)	54 (100%)	
Partial orchidectomy	21 (58.33%)	0	
Tumor length (cm)			<.001
Mean ± SD	2.82 ± 1.91	4.36 ± 2.13	
Median (range)	2.45 (0.9–11.0)	4.0 (0.5–10.0)	
Peripheral blood cells			
Neutrophil count (10^9/L)			.699
Mean ± SD	3.42 ± 1.11	3.68 ± 1.59	
Median (range)	3.18 (1.94–5.90)	3.19 (1.72–8.24)	
Lymphocyte count (10^9/L)			.880
Mean ± SD	1.98 ± 0.44	1.96 ± 0.61	
Median (range)	1.98 (1.20–2.89)	1.91 (0.70–3.80)	
Monocyte count (10^9/L)			.020
Mean ± SD	0.38 ± 0.12	0.47 ± 0.17	
Median (range)	0.38 (0.02–0.58)	0.46 (0.18–1.13)	
Red blood cell count (10^9/L)			.468
Mean ± SD	5.02 ± 0.47	4.98 ± 0.44	
Median (range)	5.06 (3.14–5.69)	5.03 (4.17–6.09)	
Platelet count (10^9/L)			.918
Mean ± SD	225.97 ± 47.07	227.41 ± 62.50	
Median (range)	224.5 (151.0–348.0)	222.5 (124.0–463.0)	
Serum indexes			
FIB level (g/L)			
Mean ± SD	2.78 ± 0.76	2.88 ± 0.95	.645
ALB level (g/L)			
Mean ± SD	46.69 ± 2.99	46.31 ± 3.81	.917
AKP level (U/L)			
Mean ± SD	78.47 ± 28.82	76.22 ± 23.72	.967
SA level (mg/dL)			
Mean ± SD	51.10 ± 5.63	53.26 ± 6.33	.169
AFU level (U/L)			
Mean ± SD	17.56 ± 6.45	18.62 ± 4.48	.480
LDH level (U/L)			
Mean ± SD	186.92 ± 29.23	202.54 ± 36.62	.035

AFU = α-L-fucosidase, AKP = alkline phosphatase, ALB = albumin, FIB = fibrinogen, LDH = lactate dehydrogenase, SA = sialic acid, SD, standard deviation, TGCT^S0^ = testicular germ cell tumor with stage S0.

### 3.2. Diagnostic value of AMC in distinguishing TGCT^S0^ from benign tumors

**Figure 2.**
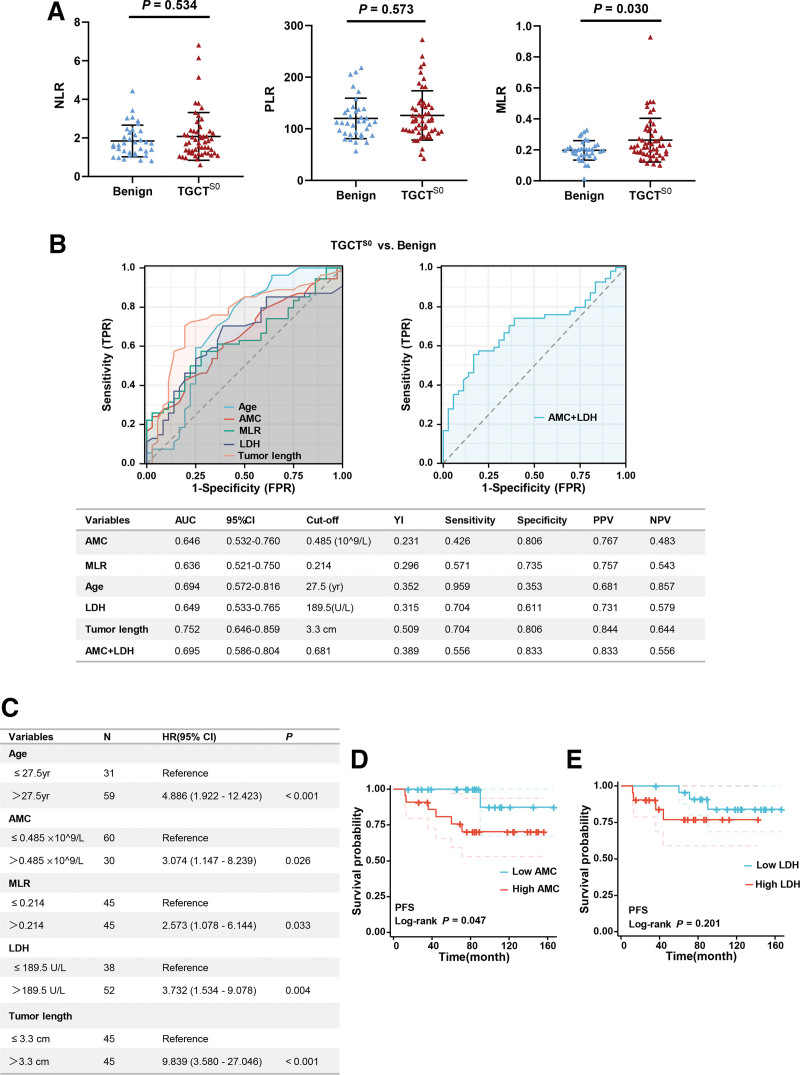
Diagnostic value of AMC in distinguishing TGCT ^S0^ from benign tumors. (A) Differences in PLR, NLR, and MLR between TGCT ^S0^ and benign tumors. (B) ROC curves for determination of cutoff value of preoperative variables regarding distinguishing TGCT^S0^ from benign tumors. The following table shows the data of ROC curves. (C) Univariate logistic regression analysis of preoperative variables on prediction of TGCT^S0^. (D) Kaplan–Meier plot showing PFS in patients with TGCT^S0^ divided into high AMC and low AMC groups. (E) Kaplan–Meier plot showing PFS in patients with TGCT^S0^ divided into high LDH and low LDH groups. AMC = absolute monocyte count, AUC = area under curve, HR = hazard ration, LDH = lactate dehydrogenase, MLR = monocyte to lymphocyte ratio, NLR = neutrophil to lymphocyte ratio, NPV = negative predictive value, PFS = progression free survival, PLR = platelet to lymphocyte ratio, PPV = positive predictive value, ROC = receiver operating characteristic, TGCT^S0^ = testicular germ cell tumor with stage S0, YI = Youden index.

Patients with TGCT^S0^ showed elevated monocyte to lymphocyte ratio (MLR) compared to patients with benign testicular tumor (0.26 ± 0.14 vs 0.20 ± 0.06, *P* = .030). However, no significant differences were observed in neutrophil to lymphocyte ratio (NLR) and platelet to lymphocyte ratio (PLR) (all *P* > .05, Fig. 2A). Figure 2B shows the ROC curves for AMC, MLR, age, LDH and tumor length regarding the prediction of TGCT^S0^. The area under the ROC curve (AUC) was 0.646 (95% CI: 0.532–0.760), 0.636 (95% CI: 0.521–0.750), 0.694 (95% CI: 0.572–0.816), 0.649 (95% CI: 0.533–0.765) and 0.752 (95% CI: 0.646–0.859), respectively. The optimal threshold value for AMC, MLR, age, LDH and tumor length with the maximum Youden index was >0.485*10^9/ L, >0.214, >27.5 year, >189.5U/L and >3.3 cm, respectively. In addition, we further evaluated the value of AMC combined with LDH for discriminating between TGCT^S0^ and benign testicular tumor, which achieved a better diagnostic efficacy (AUC = 0.695). The sensitivity, specificity, positive predictive value (PPV) and negative predictive value (NPV) for defining accuracy of the thresholds are shown in the following table in Figure 2B. Logistic regression analysis was performed to further evaluate the clinical impact of AMC, age, MLR, LDH and tumor length on the prediction of TGCT^S0^. In univariate analysis, elevated AMC and LDH were significantly associated with TGCT^S0^ compared to benign tumor (HR = 3.074, *P* = .026; HR = 3.732, *P* = .004; respectively). Moreover, tumor length was also a strong factor related to TGCT^S0^ (HR = 9.839, *P<*0.001; Fig. 2C). Furthermore, Kaplan–Meier curves revealed that patients with higher AMC level had worse progression free survival (PFS) (HR = 6.430, *P* = .047; Fig. 2D), but LDH level was not significantly associated with PFS (HR = 2.524, *P* = .201; Fig. 2E).

### 3.3. Monocyte/macrophage signature gene enrichment analysis in TGCT

**Figure 3.**
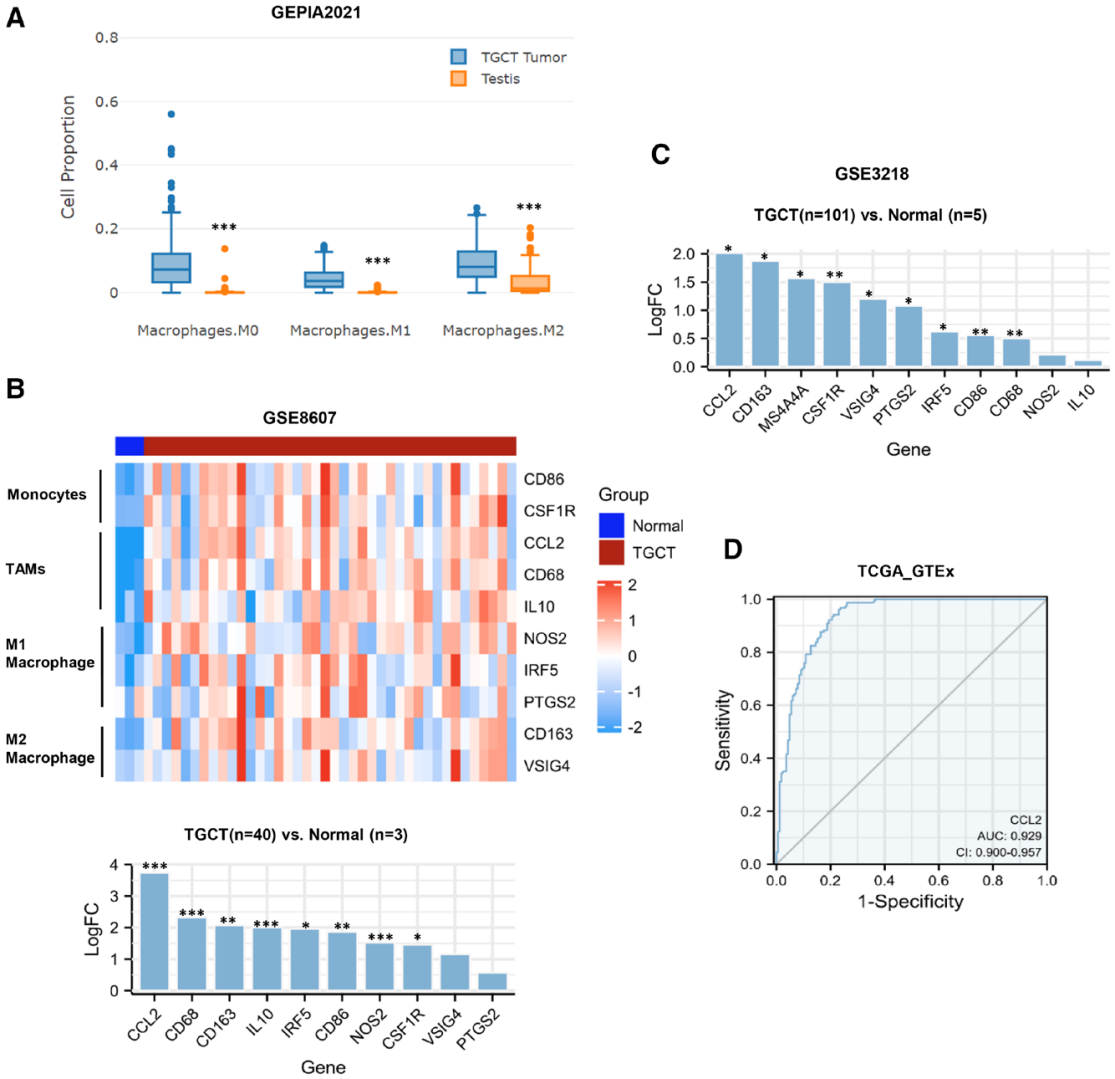
Monocyte/macrophage signature gene enrichment analysis in TGCT. (A) The proportion of different cell types of macrophage in TGCT and normal testis analyzed by GEPIA2021. (B) Top: Representative heat map of the 10 macrophage signature genes using GSE8607 data. Bottom: Bar graph showing the fold difference of 10 monocyte/macrophage signature genes between TGCT and normal tissues. (C) Bar graph showing the fold difference of 11 monocyte/macrophage signature genes between TGCT and normal tissues using GSE3218 data. (D) ROC curves of preoperative variables regarding discriminating between TGCT and normal tissues in TCGA_GTEx database. *P < .05; **P < .01; ***P < .001. CCL2 = C-C motif chemokine ligand 2, GEPIA2021 = Gene Expression Profiling Interactive Analysis 2021, GTEx = The Genotype-Tissue Expression, LogFC = log_2_(fold change); ROC = receiver operating characteristic, TAMs = tumor-associated macrophages, TCGA = the Cancer Genome Atlas, TGCT = testicular germ cell tumor.

In the circulatory system, monocytes penetrate the wall of the bleeding tube and become macrophages in the tissue. In the GEPIA2021 database, we found that the proportion of different types of macrophages in TGCT tissue was significantly higher than that in normal testicular tissue (all *P < *.001, Fig. 3A). To identify the expression differences of marker genes of macrophages between TGCT and normal tissues, we performed targeted gene expression analysis using 2 gene expression omnibus data (GSE8607 and GSE3218). Among the identified genes, tumor-associated macrophages (TAMs) signature gene-CCL2 was increased in TGCT and the fold difference was the most significant in both 2 data sets as shown in Figure 3B and C. Therefore, we chose to focus on CCL2 as a potential TGCT gene biomarker for further study. The ROC curve indicated that CCL2 had a high diagnostic efficiency for distinguishing TGCT from normal tissues (AUC = 0.929, Fig. 3D).

### 3.4. The correlation between CCL2 and clinicopathological parameters of patients with TGCT^S0^

**Figure 4.**
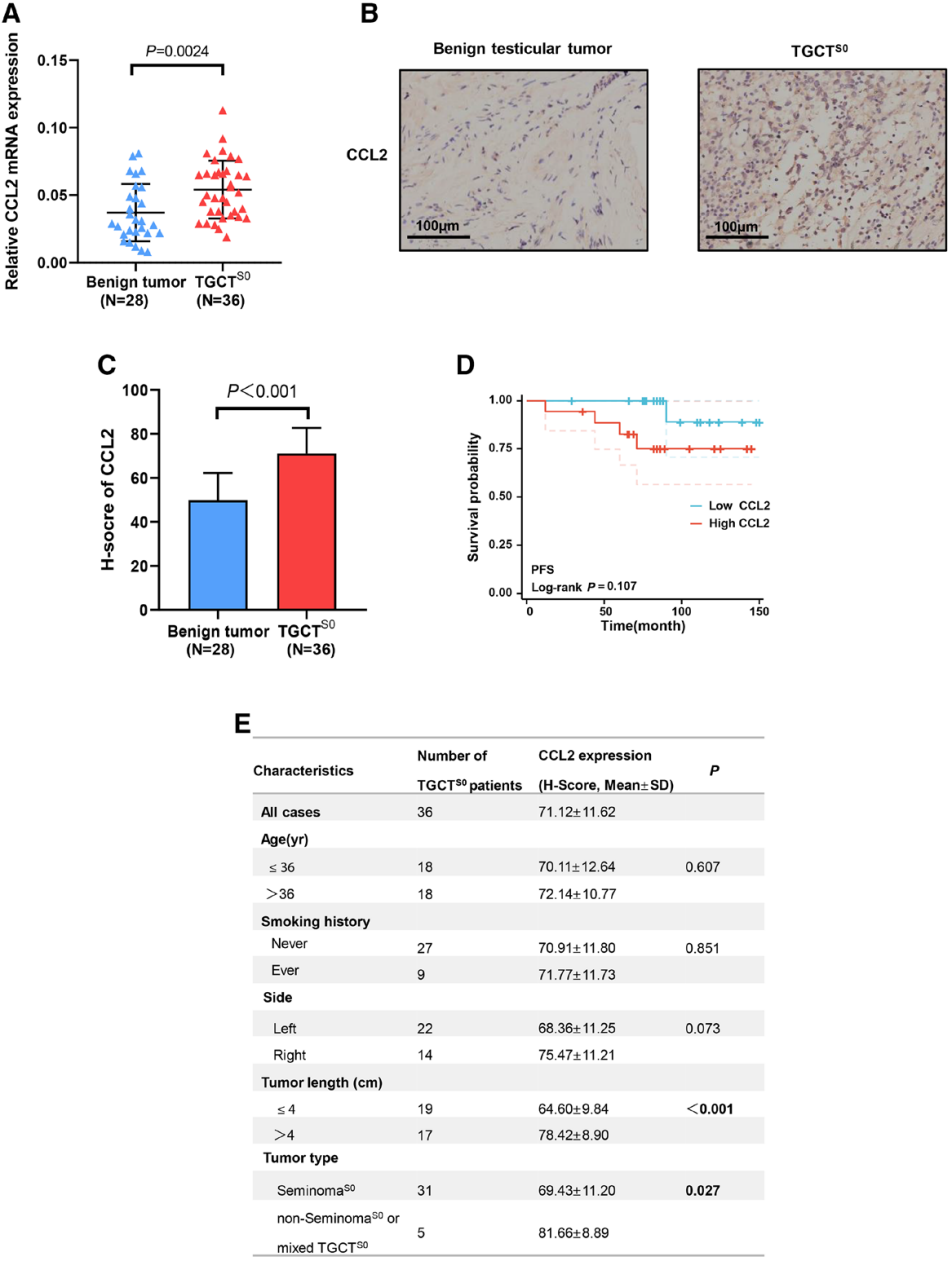
Expression of CCL2 in TGCT^S0^. (A) The expression of CCL2 mRNA expression in benign testicular tumor (n = 28) and TGCT^S0^ (n = 36). (B) The expression of CCL2 protein examined in benign testicular tumor (n = 28) and TGCT^S0^ (n = 36) by IHC analysis. Scale bars represent 100 μm and (C) H-scores (right panel) were indicated. (D) Kaplan–Meier plot showing PFS in patients with TGCT^S0^ divided into high CCL2 and low CCL2 groups. (E) Correlation between CCL2 expression and clinicopathological features of patients with TGCT^S0^. CCL2 = C-C motif chemokine ligand 2, H-score = (percentage of weak intensity × 1) + (percentage of moderate intensity × 2) + (percentage of strong intensity × 3), HR = hazard ration, IHC = immunohistochemistry, PFS = progression free survival, TGCT^S0^ = testicular germ cell tumor with stage S0.

The expression level of CCL2 mRNA was significantly higher in TGCT^S0^ tissues (n = 36) compared to benign tumors (n = 28) using qRT-PCR (*P* = .0024; Fig. 4A). The IHC analysis showed that the H-score of CCL2 was significantly higher in TGCT^S0^ tissues (*P < *.001; Fig. 4B and C). Furthermore, we predicted the prognosis of CCL2 in TGCT^S0^ and found that high CCL2 expression may have a worse PFS without statistical significance (HR = 5.02, *P* = .107; Fig. 4D). Figure 4E showed the correlation between CCL2 expression and clinicopathological parameters of TGCT^S0^. The H-score of CCL2 was higher in non-Seminoma^S0^ or mixed TGCT^S0^ compared to Seminoma^S0^ (*P* = .027) and larger tumors showed higher CCL2 expression (*P < *.001).

### 3.5. Functional analyses of CCL2 in TGCT

**Figure 5.**
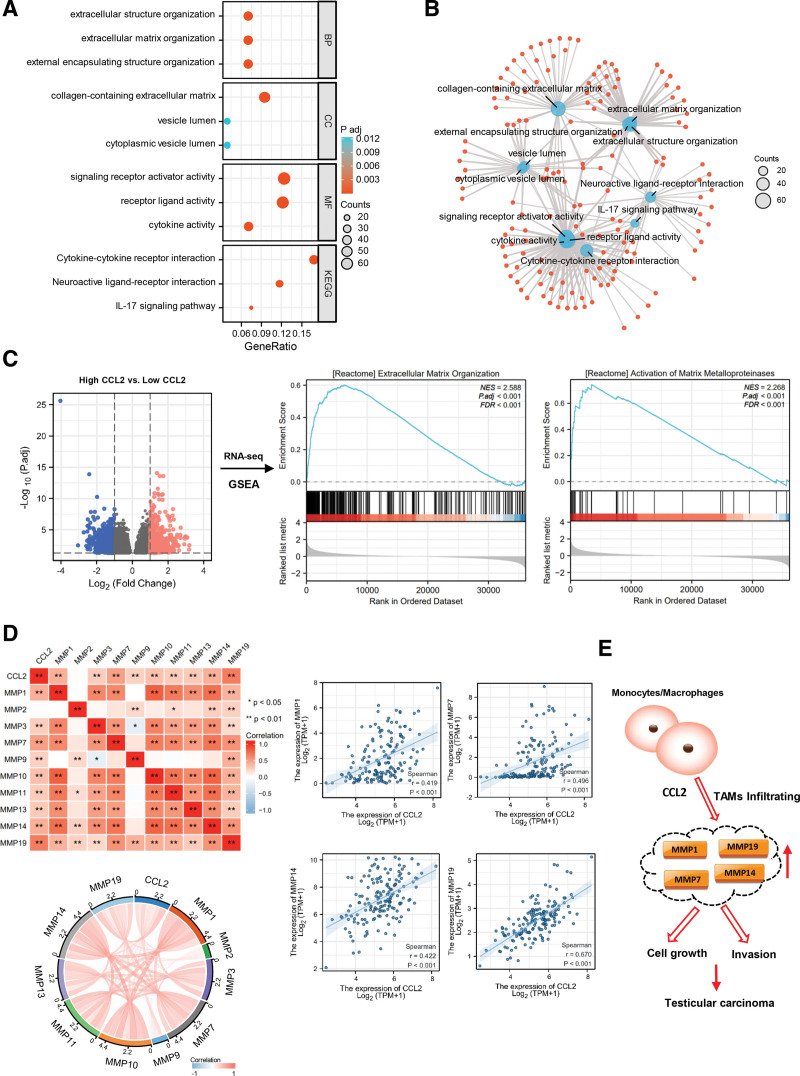
Functional analyses of CCL2 in TGCT. (A) The GO&KEGG analysis and (B) molecular network of CCL2 in TGCT. (C) GSEA analysis on High CCL2 versus Low CCL2 TGCT specimens in TCGA database. GSEA enrichment plot showing for [Reactome] Extracellular Matrix Organization and [Reactome] Activation of Matrix Metalloproteinases gene set. (D) Correlation analyses between the expression of CCL2 and MMPs expression in TGCT. (E) CCL2/MMPs mediated macrophages infiltrating promoting tumorigenesis of TGCT. BP = biological process, CC = cellular component, CCL2 = C-C motif chemokine ligand 2, GSEA = Gene Set Enrichment Analysis, GO = Gene Ontology, KEGG = The Kyoto Encyclopedia of Genes and Genomes, MF = molecular function, MMPs = matrix metalloproteinases, NES = normalized enrichment score, TAMs = tumor-associated macrophages, TCGA = the Cancer Genome Atlas, TGCT = testicular germ cell tumor.

To elucidate the possible mechanism of CCL2 in TGCT tumorigenesis, the Gene Ontology/Kyoto Encyclopedia of Genes and Genomes analysis was applied. The analysis results indicated that CCL2 plays a role in the regulation of cytokine-cytokine receptor interaction, signaling receptor activator activity, receptor ligand activity, collagen-containing extracellular matrix, extracellular structure organization, extracellular matrix organization, etc (Fig. 5A and B). Gene set enrichment analysis (GSEA) of RNA-seq from TGCT specimens of the Cancer Genome Atlas database representing major biological processes revealed increased expression of the Extracellular Matrix Organization and the Activation of Matrix Metalloproteinases gene sets (High CCL2 vs low CCL2, Fig. 5C). We further explored the correlation between CCL2 and matrix metalloproteinases (MMPs), the results showed that CCL2 mRNA expression positively correlated with MMP1, MMP3, MMP7, MMP9, MMP10, MMP11, MMP13, MMP14, and MMP19 (Fig. 5D). We hypothesized that CCL2 may promote TAMs infiltration in testicular tissue and induce tumor generation by regulating MMPs (Fig. 5E).

## 4. Discussion

In the present study, we examined preoperative peripheral AMC and other clinical features of 90 patients with benign testicular tumor or TGCT^S0^. A logistic regression analysis showed that elevated AMC level was an independent predictor of TGCT^S0^.Our results indicate that AMC is a promising biomarker for differential diagnosis of benign testicular tumor and TGCT^S0^, and CCL2 regulated TAMs infiltration and MMPs release plays a role in tumorigenesis of TGCT.

The formation of tumor is closely related to the imbalance of systemic inflammatory response.^[23]^ As an important inflammatory factor in the body, monocytes can affect the initiation, proliferation, invasion and metastasis of tumors by influencing the tumor microenvironment. Studies have shown that inflammatory cells can induce the transfer of monocytes from bone marrow to peripheral blood, and monocytes are then aggregated into tumor tissues and differentiated into TAMs, which promotes tumor angiogenesis and remodeling of tumor extracellular matrix by secreting epidermal growth factor, vascular endothelial growth factor, interleukin-6, interleukin-10 and MMPs, thus promoting the invasion and metastasis of tumor cells.^[24,25]^ In addition, TAMs can upregulate the expression of programmed cell death molecule 1 (PD-1), thus forming a local immunosuppressive microenvironment that facilitates the immune escape of tumor cells.^[26]^ In this study, the marker gene CCL2 of TAMs was significantly overexpressed in tumor tissues, suggesting that TAMs and CCL2 play an important role in the occurrence and development of TGCT.

CCL2 is a potent monocyte/macrophage chemokine, which is mainly secreted and expressed by tumor cells, and can also be generated by endothelial cells, fibroblasts and TAMs.^[27]^ It is one of the important chemokines in the tumor microenvironment and it is the main substance that determines the content of macrophages in tumors. Studies have shown that stromal cells in tumor tissues can also secrete CCL2.^[28]^ Through tumor-stromal interaction, CCL2 is responsible for recruiting macrophages to the tumor area, and may promote tumor cells to invade and metastasize to surrounding and distant tissues by secreting various enzymes to degrade extracellular matrix. MMPs are the most important proteases for extracellular matrix degradation, and there are currently 26 members, which are divided into 5 subtypes.^[29]^ Previous studies have shown that the overexpression of MMPs is closely related to tumor invasion, metastasis and poor prognosis.^[30,31]^ In our study, it was found that CCL2 was positively correlated with the expression of various MMPs, such as MMP1, MMP7, MMP14, and MMP19, suggesting that CCL2 may induce tumor cell proliferation and invasion by promoting the secretion of MMPs.

Clinically, for testicular tumor patients with negative tumor markers (Stage S0), how to distinguish the benign from the malignant is a difficult problem. Some benign tumor patients were misdiagnosed as malignancy before surgery and underwent orchiectomy. The statistical results of this study showed that nearly half of benign tumor patients (41.67%) received orchiectomy, causing irreversible physical and psychological trauma to patients of reproductive age. The current gold standard for differential diagnosis is rapid intraoperative pathology, but rapid intraoperative pathology can trigger the production of ASA, which interfere with sperm motility and fertilization after binding with sperm. In addition, ASA can also induce sperm apoptosis and promote the removal of sperm by phagocytes, thus affecting the reproductive function of the remaining testis.^[32,33]^ Therefore, it is urgent to find the new markers for the differential diagnosis of testicular tumor. Measurement of AMC, MLR and other inflammatory parameters are convenient, inexpensive and easily implemented, which does not increase the burden of patients, and is a must-check item for every patient. If multiple large sample studies can confirm and improve the value of AMC in the differential diagnosis of testicular tumors, it will also be a major breakthrough and may be widely used in clinical practice to improve the diagnostic efficiency of testicular malignant tumors and reduce unnecessary surgical injuries.

A few limitations of the current study should be noted. First, because of the retrospective design of this study and the limited number of patients, unknown sources of bias may exist in the findings. Furthermore, this investigation of testicular tumors was performed in a single institution; thus, the generalizability of the results may be limited. Finally, further investigations are warranted to elucidate the carcinogenic mechanism of CCL2 in TGCT.

## 5. Conclusions

The present study demonstrates that elevated preoperative AMC level may serve as a predictor of higher risk of tumor marker-negative TGCT, and CCL2 plays an important role in TAMs infiltration and MMPs secretion to induce tumorigenesis of TGCT.

## Author contributions

**Conceptualization:** Yidong Fan, Jikai Liu.

**Data curation:** Li Cao, Jiajia Sun, Shuo Pang.

**Formal analysis:** Li Cao, Qinzheng Chang.

**Funding acquisition:** Jikai Liu.

**Investigation:** Li Cao, Qinzheng Chang.

**Methodology:** Shuo Pang, Jikai Liu.

**Supervision:** Yidong Fan, Jikai Liu.

**Validation:** Qinzheng Chang, Jiajia Sun.

**Writing – original draft:** Li Cao, Jikai Liu.

**Writing – review & editing:** Jiajia Sun, Jikai Liu.
